# The Influence of Obstructive Sleep Apnea on Post-Stroke Complications: A Systematic Review and Meta-Analysis

**DOI:** 10.3390/jcm13185646

**Published:** 2024-09-23

**Authors:** Nithin Kurra, Nikhila Gandrakota, Manju Ramakrishnan, Kavya Sudireddy, Naga Vijaya Lakshmi Divya Boorle, Dinesh Jillella

**Affiliations:** 1 Department of Neurology, University of Nebraska Medical Center, Omaha, NE 68198, USA; 2 Department of Family & Preventive Medicine, Emory University School of Medicine, Atlanta, GA 30322, USA; nikhila.gandrakota@emory.edu; 3 Rollins School of Public Health, Emory University, Atlanta, GA 30322, USA; manju.ramakrishnan@emory.edu; 4 Department of Internal Medicine, Chan Medical School, University of Massachusetts, Worcester, MA 01655, USA; kavya.sudireddy@umassmemorial.org; 5 School of Medicine, Wayne State University, Detroit, MI 48201, USA; divya.boorle@wayne.edu; 6 Department of Neurology, Emory University School of Medicine, Atlanta, GA 30322, USA; dinesh.jillella@emory.edu

**Keywords:** obstructive sleep apnea, sleep-disordered breathing, apnea–hypopnea, post-stroke complication, stroke

## Abstract

**Objectives:** Evidence shows that obstructive sleep apnea (OSA) is associated with the development of stroke. This study investigates the relationship between OSA and post-stroke complications, addressing the limited data on how OSA influences the severity and development of these complications through a systematic review of existing literature. **Methods:** Data was collected from PubMed, Web of Science, and Scopus databases up to December 2023. Studies meeting the inclusion criteria were selected, and statistical analyses were performed using Review Manager 5.4.1. A random-effects model was used for pooling data with heterogeneity, and findings were presented using standard ratios with 95% confidence intervals. **Results:** The analysis included nine studies. Stroke patients with OSA did not show a significantly higher risk of post-stroke complications, which include mild cognitive impairment, dementia, insomnia, fatigue, reduced sleep quality, depression, anxiety, recurrent strokes, and death, compared with those without OSA (RR = 1.05, 95% CI 0.97 to 1.13). However, patients with high stroke severity and OSA had a slightly higher risk of post-stroke complications (RR = 1.06, 95% CI 1.01 to 1.12). **Conclusions:** This systematic review and meta-analysis suggests that OSA confers a higher risk of post-stroke complications in patients with high stroke severity. Further studies are required to explore the impact of OSA on post-stroke complications.

## 1. Introduction

Obstructive sleep apnea (OSA) is a common type of sleep-disordered breathing (SDB) characterized by the partial or complete collapse of the pharyngeal airway and a subsequent reduction in oxygenation [1]. Individuals with OSA experience interrupted sleep, snoring, daytime sleepiness, gasping, choking (due to apneic episodes), and morning headaches [2]. Moreover, undiagnosed and/or untreated OSA has been associated with various negative outcomes, including cardiovascular, mental health, and metabolic disorders [2,3].

Stroke, a leading cause of morbidity and mortality worldwide, is influenced by various modifiable risk factors such as hypertension, diabetes, dyslipidemia, and infections [4]. Recent studies have indicated a potential association between obstructive sleep apnea and many of these risk factors, which can impact an individual’s predisposition to stroke. Furthermore, evidence suggests an independent bidirectional relationship between stroke and OSA [5]. The characteristic intermittent apneic/hypoxemic episodes observed in OSA lead to fluctuations in blood pressure and physiological stress-induced sympathetic activation, thereby establishing a direct or indirect association between OSA and stroke [6].

Despite an abundance of studies establishing obstructive sleep apnea (OSA) as a risk factor for cerebrovascular disruption, there remains a scarcity of research examining the implications of a history of sleep apnea in the post-stroke period. While physical disabilities resulting from a cerebrovascular accident are well-documented, it is important to recognize that stroke patients often experience a myriad of complications, including recurrent strokes, seizures, cardiovascular effects, insomnia, depression, mood changes, cognitive impairment, and, in severe cases, increased mortality [7].

Emerging evidence suggests that OSA not only contributes to the development of these complications but also influences their prognosis, with the severity of OSA exacerbating their impact. In a recent hospital-based study conducted by Li, C., et al. [8], a significant association was found between the severity of obstructive sleep apnea and post-stroke depression after 3 months. Kaneko, Y., et al. [9], demonstrated a higher prevalence of functional impairment among stroke patients with concomitant obstructive sleep apnea. This association suggests that OSA may contribute to a prolonged period of rehabilitation, further hindering the recovery and functional outcomes of stroke survivors. These findings underscore the potential role of OSA in exacerbating post-stroke complications.

While the relationship between OSA and stroke is sufficiently established, further research is required to fully understand how OSA affects post-stroke outcomes and complications. Through our study, we aim to investigate the association between obstructive sleep apnea and post-stroke complications. By recognizing the impacts of OSA, targeted interventions can be developed to improve prognoses and to formulate a more comprehensive management plan in clinical practice while improving the overall outcome and quality of life in stroke patients.

## 2. Methods

### 2.1. Search Strategy and Selection Criteria

To obtain pertinent clinical data from the published literature up to 31 December 2023, a thorough search strategy employing the PubMed, Web of Science, and Scopus databases was developed by N.C.K, N.G., M.R., N.V.L.D.B, and K.S. Studies were chosen and data were extracted by two separate authors, N.G. and N.C.K. The final decision on eligibility was made after discussions among N.C.K., N.G., M.R., N.V.L.D.B., and K.S. To include all relevant data fully and accurately, this research used broad inclusion criteria. This review was not registered in any prospective systematic review registry.

### 2.2. Data Extraction

The following information was extracted from each study: (1) Study identity; (2) Number of stroke patients that had OSA; (3) Number of stroke patients that were free from OSA; (4) Presence of post-stroke complications in both groups; (5) Interventions and outcomes; (6) Severity of stroke.

### 2.3. Study Inclusion

#### 2.3.1. Inclusion Criteria

The main inclusion criterion for this study was that participants were diagnosed with obstructive sleep apnea (OSA). Additionally, only studies that reported or provided information on OSA were included. Furthermore, only studies that specifically investigated the association between OSA and post-stroke complications were included in the analysis (see Table 1). Nine studies were selected for the meta-analysis (Zhang 2022, et al. [10], Aaronson, et al. [11], Zhu, et al. [12], Zhang 2017, et al. [13], Slonkova, et al. [14], Meng, et al. [15], Li 2019, et al. [16], Chen and Marsh [17], and Li 2020, et al. [8]) [8,10,11,12,13,14,15,16,17].

#### 2.3.2. Study Exclusion

Studies that did not specifically address post-stroke OSA or that lacked sufficient information on OSA status or post-stroke complications were excluded. Additionally, we excluded case reports, case series, systematic reviews, meta-analyses, and studies where outcomes were incomplete or improperly reported. Thirty-two studies were excluded from the meta-analysis due to reporting outcomes that did not align with the research focus or employing study designs that did not meet the inclusion criteria.

### 2.4. Statistical Analysis

**Figure 1:**
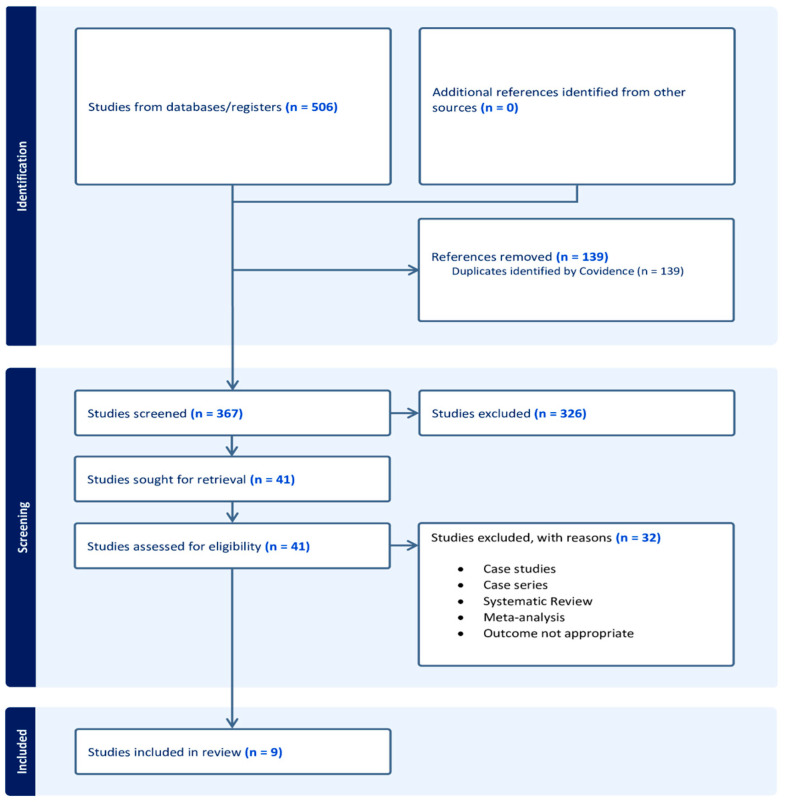
PRISMA flow diagram.

To demonstrate the relationship between OSA and post-stroke issues, our study considered two groups of stroke patients: those with OSA and those without OSA. To summarize the relationship between OSA and stroke patients, we utilized a Mantel–Haenszel random-effects meta-analysis. The proportion of patients who experienced post-stroke complications was calculated, along with their 95% confidence intervals (CIs). For all meta-analyses, prevalence estimates were converted to logits to enhance their statistical characteristics and subsequently back transformed. Using RevMan 5.4, I^2^ statistics evaluated the variability of prevalence estimates among studies. Obstructive sleep apnea, according to our hypothesis, would raise the likelihood of post-stroke problems. As a result, it is anticipated that for each study accepting this hypothesis (favoring OSA patients with worse outcomes), larger squared effect-size would be shown on the left of the forest plot. The chi statistic and the inconsistency index (I^2^) statistics were also shown in the forest plot to determine whether the studies were heterogeneous or homogeneous. Low heterogeneity was indicated by an I^2^ value of less than 50%. Mild variability was evident in the range from fifty to seventy-five. At the same time, I^2^ value of over 75 displayed a high degree of heterogeneity. Figure 1 summarizes the literature search and review strategy using a PRISMA flow diagram.

## 3. Results

**Figure 2:**
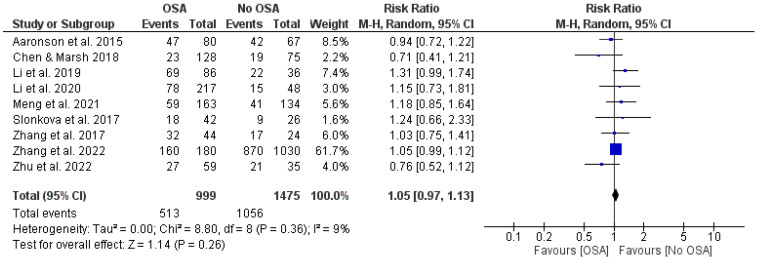
Forest plot showing the association of OSA with prevalence of post-stroke complications [8,10,11,12,13,14,15,16,17].

The pooled effect for the association (RR = 1.05, 95% CI 0.97 to 1.13, I^2^ = 9%; Figure 2) suggests that stroke patients with OSA have a trend toward a higher likelihood of having post-stroke complications (mild cognitive impairment, dementia, insomnia, fatigue, sleep quality, post-stroke depression, anxiety, recurrent strokes) compared with those stroke patients who do not have OSA, although that was not statistically significant in our analysis. The percentage of heterogeneity across studies (I^2^ = 9%) was low and not statistically significant (*p* = 0.36), and the Z-score statistics were normally distributed (Z score = 1.14).

**Figure 3:**
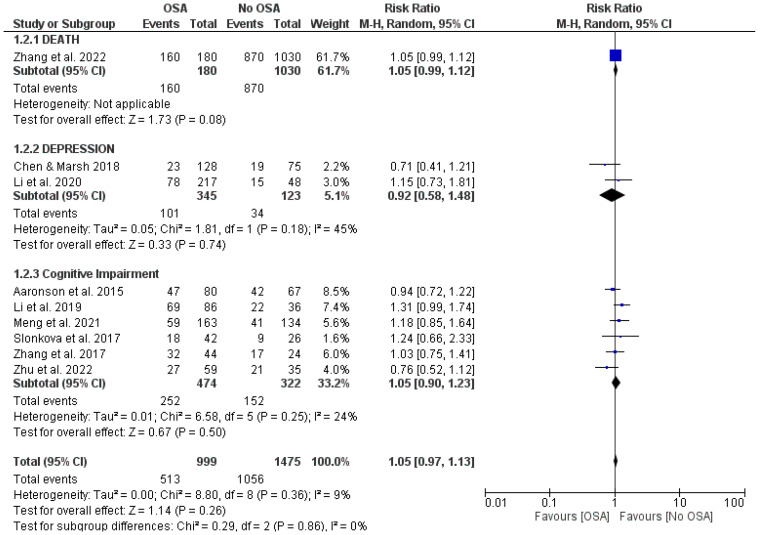
Forest plot of the impact of OSA on different post-stroke complications [8,10,11,12,13,14,15,16,17].

One study (Zhang et al. 2022 [10]) reported mortality (death) as a complication. This study did not find a statistically significant difference in the prevalence of death among stroke patients with OSA compared with those without OSA (RR = 1.05, 95% CI 0.99 to 1.12; Figure 3), despite a slight trend toward a higher prevalence in the OSA group.

Two studies (Li et al. 2020 [8], Chen and Marsh 2018 [17]) examined depression as a complication of stroke. Both studies showed no significant association between OSA and the risk of depression in stroke patients compared with controls (RR = 0.92, 95% CI = 0.58 to 1.48, I² = 45%; Figure 3).

Cognitive impairment was assessed in six studies (Aaronson, et al. [11], Zhu, et al. [12], Zhang, et al. [13], Slonkova, et al. [14], Meng, et al. [15], Li, et al. [16]). These did not demonstrate a significantly increased risk in stroke patients with OSA compared with those without (RR = 1.05, 95% CI −0.90 to 1.23, I^2^ = 24%; Figure 3), although, again, a trend toward increased risk was observed.

**Figure 4:**
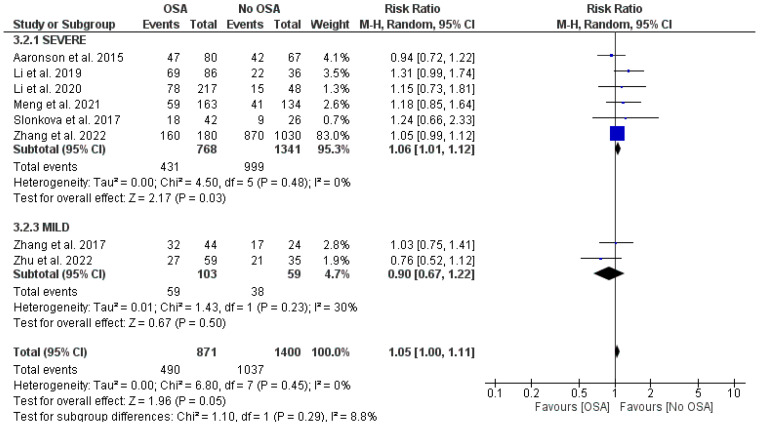
Forest plot showing the association of OSA according to the severity of stroke [8,10,11,12,13,14,15,16].

The stroke severity in patients was categorized into three classes (mild, moderate, and severe). Chen and Marsh [17] did not report the stroke severity, while Zhang 2017 [13] and Zhu 2022 [12] reported the level of severity as mild, but they did not reveal any significant association between OSA and post-stroke complications when the stroke severity was categorized as mild (RR = 0.90, 95% CI −0.67 to 1.22, I^2^ = 30%; Figure 4) [12,13,17]. Six studies reported the severity level as severe. These studies revealed that among stroke patients with high severity, patients with OSA have a higher risk of post-stroke complications than those patients without OSA (RR = 1.06, 95% CI −1.01 to 1.12; Figure 4) [8,10,11,14,15,16].

## 4. Discussion

Obstructive sleep apnea is an important risk factor associated with the development of stroke. Previous studies primarily focused on establishing evidence of OSA as a comorbid condition contributing to the evolution of a cerebrovascular accident. There is limited data regarding its role in the development of the potential issues following a stroke. In this meta-analysis, we analyzed existing evidence from nine studies to assess the post-stroke complications in patients diagnosed with OSA and evaluated their association based on the severity of the stroke. Our pooled results suggest a nominally increased risk of post-stroke complications in patients with OSA and stroke but a significantly increased risk in patients with severe stroke. The predominant outcomes observed in these studies were cognitive impairment, depression, and death.

The most observed post-stroke outcome was cognitive impairment among those with obstructive sleep apnea, which was demonstrated in seven studies. Although there may be several factors like cardiovascular dysfunction, insomnia, and genetic susceptibility that can contribute to the complexity of such neuropsychiatric consequences, especially in those with stroke, sleep apnea must be recognized as an important instigating factor for hypoxia-induced effects [10,18,19]. Zhu et al., based on the apnea–hypopnea index (AHI) for assessing OSA, reported that there was a significant association between higher AHI scores and the loss of cognition. This also correlated with the low oxygen saturation (SpO2) observed in patients with the presence of OSA compared with those without [12].

Previous literature suggests that apnea–hypopnea episodes induce a state of chronic intermittent hypoxia, which triggers oxidative stress as well as neuroinflammation in the body, resulting in cognitive decline [20,21]. Furthermore, Bubu et al. demonstrated that obstructive sleep apnea is indirectly responsible for an accelerated accumulation of amyloid beta peptide (Aβ) and tau accumulation. This is prudent to note when considering the long-term consequences of a history of obstructive sleep apnea prior to the incidence of stroke [22]. In a hospital-based study conducted by Kang et al., the presence of Aβ deposits was determined to instigate the course of post-stroke cognitive impairment. They reported that Aβ positivity showed a greater post-stroke cognitive decline after 1 year [23]. Our study reinforces the need for early screening of OSA to initiate the management of patients with treatment like positive airway pressure (CPAP) which has been known to provide symptomatic relief, reduce severity, and improve the affected neurocognition in CVA patients [24,25].

Li et al. conducted a hospital-based study to investigate the connection between post-stroke depression (PSD) and OSA. The severity of OSA, which was determined by the AHI and the oxygen desaturation index (ODI), and its relationship with post-stroke depression, which was evaluated using the Hamilton Depression Scale (HAMD) and the Chinese Structured Clinical Interview, were analyzed. They determined that there was a correlation between the OSA severity and the establishment of PSD [8].

During the literature review, our investigation revealed a paucity of studies that examined this relationship. Previous studies discussed several mechanisms involved in the onset of depression following a stroke [8,26,27]. In OSA, there are repeated fluctuations observed in the cerebral blood flow velocity with every apnea–hypopnea episode. As a result, the chronic hypoperfusion induces hypoxemic–ischemic injury, endothelial damage disrupting the vasodilator nitric oxide (NO) production, and the repeated deoxygenation and re-oxygenation damaging the blood–brain barrier and unfolding neuroinflammation, all of which precipitate the evolution of cerebral small-vessel disease, leading to vascular depression [8,26,27]. These pathophysiological mechanisms and biological processes involved in the presence of other comorbidities of stroke can interact to increase the risk of neuropsychiatric complications.

The study conducted by Zhang et al. examined the effects of sleep disorders and OSA on neurological function and overall mortality after a stroke. The findings suggested that individuals with OSA have a higher risk of all-cause mortality, especially in patients with severe OSA [10]. According to previous evidence, OSA is independently linked to the development of several cardiovascular complications, including hypertension, atrial fibrillation, coronary heart disease, heart failure, etc., and all-cause mortality due to various pathophysiological processes, with the most prominent mechanisms involving an autonomic dysregulation and oxidative stress [28,29,30]. Although there is a dearth of data supporting the insights from this study, these results emphasize the necessity for additional research to better comprehend the connections and mechanisms involved and to increase the evidence supporting a deeper understanding of the relationship between OSA and complications during the post-stroke period.

To our knowledge, our study is one of the earliest to highlight valuable insights on OSA and post-stroke events. However, while it brings attention to the importance of future research considerations, some of the limitations require disclosure. All studies included in this meta-analysis have identified various study cohorts. Furthermore, it is also prudent to acknowledge that a diverse set of criteria was used to categorize the population into distinct categories based on stroke severity, namely mild, moderate, and severe. Further research is required to explore OSA and its effect in the aftermath of a stroke, which would improve the generalizability of the results.

## 5. Conclusions

OSA during the post-stroke period is an important comorbidity that can increase the risk of mortality, cognitive decline, depression, and poor functional outcomes, especially in severe stroke patients. While the exact mechanisms involved in the associations are unclear, these observational findings need to be further investigated using large-scale experimental study designs with diverse populations. Based on the current evidence, early assessment of OSA with necessary interventions would help reduce the post-stroke disease burden, inform future interventions, and improve post-stroke recovery and management outcomes.

## Figures and Tables

**Table 1:** Summary of all the included studies.

S.No.	First Author	Title	DOI	Year	Journal	Country	N	Time Period of Investigation	Population	Study Design	Intervention	Comparison	Outcome	*p*-Value for Stroke Risk
1	Yajing Zhang [10]	Relationship between sleep disorders and the prognosis of neurological function after stroke	https://doi.org/10.3389/fneur.2022.1036980 (accessed on 20 September 2024)	2022	*Frontiers in Neurology*	China	1542	3 months, 6 months, and 4 years after stroke	age 61.58 ± 10.71	Prospective cohort study	Patient personal histories were recorded; MMSE (mini-mental state examination), MoCA (Montreal Cognitive Assessment), HAMD (Hamilton Depression Scale), National Institutes of Health Stroke Scale (NIHSS) score, mRS (Modified Rankin Scale), BI (Barthel Index), PSQI (Pittsburgh Sleep Quality Index), ESS (Epworth Sleepiness Scale), Berlin questionnaire, and nocturnal TST (Total sleep time) were assessed before discharge, and at 3 months, 6 months, and 4 years after stroke.	None	Low sleep quality (OR 2.019, 95% CI 1.199–3.398, *p* = 0.008), nocturnal TST (<7 h) (OR 4.060, 95% CI 1.494–11.034, *p* = 0.006), and nocturnal TST (>8 h) (OR 5.928, 95% CI 2.134–16.464, *p* = 0.001) were risk factors for poor neurological function recovery at 3 months after stroke. Nocturnal TST (<7 h) (OR 13.042, 95% CI 2.576–66.027, *p* = 0.002) and nocturnal TST (>8 h) (OR 11.559, 95% CI 2.108–63.390, *p* = 0.005) were risk factors for poor neurological function at 6 months after stroke. Nocturnal TST (<7 h) (OR 2.668, 95% CI 1.250–5.698, *p* = 0.011) and nocturnal TST (>8 h) (OR 2.516, 95% CI 1.080–5.861, *p* = 0.033) were risk factors for poor neurological function at 4 years after stroke. High risk of OSA (OR 1.582, 95% CI 1.244–2.012, *p* < 0.001) was a risk factor for all-cause death in patients followed up for 4 years after stroke	*p* < 0.001
2	Ruo-lin Zhu [12]	Obstructive sleep apnea is associated with cognitive impairment in minor ischemic stroke	https://doi.org/10.1007/s11325-022-02575-5 (accessed on 20 September 2024)	2022	*Sleep and Breathing—International Journal of the Science and Practice* of *Sleep Medicine*	China	156		18 to 75 years of age;	Case–control study	Patients with an initial diagnosis of ischemic stroke were included in the study. Overnight polsomnography was conducted to assess OSA, and a set of neurological tests were performed to evaluate cognitive function.	Stroke patients without obstructive sleep apnea	- Out of 94 patients, 35 had no OSA, 32 had mild OSA, and 27 had moderate to severe OSA. - Based on the analysis, risk factors associated with the onset of OSA in stoke patients include gender, hypertension, and smoking (odds ratio (OR) = 0.17, 95% CI 0.04 to 0.74, *p* = 0.02; OR = 4.61, 95% CI 1.45 to 14.69, *p* = 0.01; and OR = 9.18, 95% CI 2.32 to 36.28, *p* = 0.00, respectively). - When compared to no or mild OSA group, the moderate-to-severe OSA group performed worse on the Chinese version of the Auditory Verbal Learning Test (CAVLT)—Recognition (*p* < 0.001), Digital Span Test (DST)—Backward (*p* < 0.001), Montreal Cognitive Assessment (MoCA) (*p* < 0.001), and Stroop Color and Word Test (SCWT)—Interference (*p* < 0.001).	
3	Jie Li [16]	Cognitive impairment and sleep disturbances after minor ischemic stroke	https://doi.org/10.1007/s11325-018-1709-4 (accessed on 20 September 2024)	2019	*Sleep and Breathing*	China	86	Within 14 days after minor stroke	29–82 years	Prospective observational study	Polysomnography (PSG) and cognitive assessments were performed within 14 days of minor stroke. OSA was defined as an apnea– hypopnea index (AHI) > 5 events/h. - An MRI brain was performed within 7 days after stroke, and the severity of leukoaraiosis was graded using the Fazekas scale. - Neuropsychological assessment was performed on the second day of PSG examination using MMSE and MoCA (Beijing Version) tests to assess the five domains (attention, executive function, language, memory and visuospatial function) of cognitive function.	Partner or friends of subjects were recruited as healthy controls.	- OSA was noted in 74.4% of patients with cognitive impairment, and the majority of them (60.9%, 39/64) were categorized as having mild to moderate OSA. - Prevalence of PSCI was 81.4% (70/86) among stroke patients. Across the five cognitive domains, impairments in attention and working memory (87.1%, 61/70) and executive function (84.3%, 59/70) were the most common, whereas language impairment was the least common (28.6%, 20/70). The - prevalence of visuospatial function and memory impairment was 61.3 and 83.8%, respectively. - PSCI patients showed a higher prevalence of obstructive sleep apnea (50.0 vs. 80.0%, *p* = 0.030) and shorter total sleep time (435.1 ± 104.0 vs. 347.3 ± 98.1 min, *p* = 0.002).	*p* < 0.030
4	Yan Zhang [13]	Obstructive sleep apnea exaggerates cognitive dysfunction in stroke patients.	https://doi.org/10.1016/j.sleep.2016.11.028 (accessed on 20 September 2024)	2017	*Sleep Medicine*	China	44	6 weeks after stroke	30–65 years	Cross-sectional study design	All subjects underwent overnight polysomnography testing and received prior evaluation for sleep and cognitive function using the Epworth sleepiness scale (ESS), the Chinese version of the Minimental state examination (MMSE), and a prospective memory test.	Non- OSA stroke patients served as control	- OSA and stroke were found to be two independent predictors for the loss of both time- and event-based prospective memory. - The coefficients of stroke and OSA were −8.16 and −2.68 for the - prediction of time-based prospective memory and −5.51 and −1.38 for event-based prospective memory, respectively; *p* < 0.01.	*p* < 0.01
5	Jana Slonkova [14]	Spontaneous improvement in both obstructive sleep apnea and cognitive impairment after stroke.	https://doi.org/10.1016/j.sleep.2016.11.024 (accessed on 20 September 2024)	2017	*Sleep Medicine*	Czech Republic	68	Up to 72 h after stroke	>18 years	Prospective cohort study	Stroke patients were recruited and followed for 12 months to observe the incidence and evolution of OSA. Sleep cardiorespiratory polygraphy (PG) was performed at 72 h, 7 days, 3 months, and 12 months after stroke; Cognitive function was assessed using the revised Addenbrooke’s Cognitive Examination (ACE-R) at 3 months and 12 months; Post-stroke functional disabilities were measured with the NIHSS and modified Rankin Scale (mRS) at entry, 3 months, and 12 months and with the Barthel Index at 3 months and 12 months.	Patients with (apnea–hypopnea index) AHI < 5.	61.8% (n = 42) subjects were disgnosed with OSA. Cognitive function was improved in the OSA group at the one-year follow up. This is reflected by the reduction in total ACE-R scores at 3 months (*p* = 0.005) and 12 months (*p* = 0.004). A significant difference is noted between OSA and non-OSA groups for subsets of memory, verbal fluency, and visual–spatial abilities (*p* ˂ 0.001).	*p* < 0.005
6	Justine A. Aaronson [11]	Obstructive Sleep Apnea is Related to Impaired Cognitive and Functional Status after Stroke.	https://doi.org/10.5665/sleep.4984 (accessed on 20 September 2024)	2015	*Sleep*	Netherlands	147	Admission to the neurorehabilitation unit between 1 and 16 weeks after stroke	18–85 years	Case–control study	Sleep assessments were carried out using standardized pulse oximetry and overnight cardiorespiratory polygraphy. The oxygen desaturation index (ODI) and apnea–hypopnea index (AHI) were used to determine cases and controls. Cognitive and functional status was determined using a battery of neuropsychological tests within 4 weeks of admission. These include the following: (1) Psychomotor Vigilance Task to test for vigilance and reaction time; (2) DKEFS Trail-Making Test for visual attention and mental flexibility, (3) d2 Test of Attention; (4) Rey Auditory Verbal Learning Test for verbal memory; (5) WAIS-III Letter–Number Sequencing to test working memory; (6) Tower of London for the assessment of executive functioning; (7) Category Fluency to assess verbal fluency; (8) Bells Test, a test for visuoperception and visual neglect; (9) Finger Tapping Test to assess psychomotor ability; and (10) WAIS-III Matrix Reasoning.	Patients with a oxygen destauration index (ODI) < 5 or apnea–hypopnea index (AHI) < 15 were enrolled as control group.	Based on the study findings, there is a significant difference between the OSA and non-OSA groups in both cognitive and functional measures. Effect size for functional independence was found to be largest. OSA patients had poor performance in the domains of attention, executive function, visuoperception, psychomotor ability, and intelligence. There was no significant difference in the domains of vigilance, memory, working memory, and language between groups.	
7	Chuanyou Li [8]	Association between obstructive sleep apnea and risk of post-stroke depression: A hospital-based study in ischemic stroke patients.	https://doi.org/10.1016/j.jstrokecerebrovasdis.2020.104876 (accessed on 20 September 2024)	2020	*J Stroke Cerebrovasc Dis*	China	265	2017–2018	>18 years	Prospective cohort study	All participants underwent polysomnography (PSG) examination during hospitalization using comprehensive portable devices. Apnea–hypopnea index (AHI) calculated by evaluating the average of the total number of obstructive apneas and hyponeas per hour of sleep. Oxygen desaturation index (ODI) assessed by calculating the number of oxygen desaturation values greater than 3% per hour of sleep. The Hamilton Depression Scale (HAMD) was used in the psychological assessment for depression. If >7, the Chinese Structured Clinical Interview for DSM-IV was used to conclude a diagnosis of post-stroke depression (PSD).	Patients without OSA	There was a significant correlation between reduced OSA severity and PSD at 3 months (*p* = 0.003). At 3 months, participants with PSD exhibited higher OSA severity (*p* = 0.004) and elevated AHI levels (*p* = 0.001) compared with those without PSD. Severe OSA significantly increased the risk of PSD (OR, 3.86; 95% CI, 1.60–9.33; *p* = 0.003) compared with patients without OSA, a finding that persisted even after adjusting for multiple factors like age, gender, white matter lesions, education status, hs-CRP levels, arousal index, and ODI.	*p* < 0.003 *p* < 0.004
8	Yuan-Yuan Meng [15]	Longitudinal cognitive dysfunction in patients with obstructive sleep apnea syndrome after transient ischemic attack.	https://doi.org/10.1097/CM9.0000000000001428 (accessed on 20 September 2024)	2021	*Chin Med J (Engl)*	China	163	2012–2017	>45 years		Baseline neuropsychological assessment, basic clinical features, vascular risk factors, hospital anxiety and depression scale (HADS), and MRI were evaluated within 7 days after admission at the 6-month and 2-year follow-up. All patients were scored according to the ABCD2 scoring method.	Subjects with moderate and severe OSA but without TIA	No significant difference was found between OSA patients with and without TIA in age, sex, education level, sleep parameters, mean score of the HADS-D, and presence of vascular risk factors (*p* > 0.05). For OSA patients with TIA, the function of the cognitive domain, including executive function (*p* < 0.001), attention (*p* < 0.001), and information processing speed (*p* < 0.001), decreased continuously at baseline, and at the 6-month and 2-year follow-up, whereas the other cognitive functions remained stable. There was a significant association of a TIA event with a decline in executive function (odds ratio (OR) = 0.046, 95% confidence interval (CI): 0.025–0.084; *p* < 0.001), attention (OR = 4.112, 95% CI: 2.971–5.692; *p* < 0.001), and information processing speed (OR = 7.258, 95% CI: 4.768–11.048; *p* < 0.001) in OSA patients.	*p* < 0.001
9	Karen Chen [17]	Chronic post-stroke fatigue: It may no longer be about the stroke itself.	https://doi.org/10.1016/j.clineuro.2018.09.027 (accessed on 20 September 2024)	2018	*Clin Neurol* *Neurosurg*	United States	203	FACIT scale was administered within 6 months post-stroke and re-administered >6 months post-infarct	>18 years old	Prospective study	Patients presenting with acute stroke were seen in follow-up within 6 months of the infarct and administered the Functional Assessment of Chronic Illness Therapy (FACIT) fatigue scale to evaluate PSF. It was also re-administered >6 months post-infarct. Demographics, stroke characteristics (NIH Stroke Scale (NIHSS), infarct size and location), medical comorbidities, and outcomes (modified Rankin Scale (mRS)) were also recorded. Regression analyses were used to determine factors associated with FACIT scores and PSF at each time point.	None	203 patients were administered the FACIT a mean 1.6 months post-stroke; 128 underwent re-administration (mean 13.9 months post-event). In adjusted models, stroke severity (follow-up NIHSS (*p* < 0.001), mRS (*p* = 0.005)) and posterior circulation localization (*p* = 0.012) were associated with lower FACIT scores (increased fatigue) in the subacute setting. Medical comorbidities (hypertension (*p* = 0.024), obstructive sleep apnea (*p* = 0.020) and medication use (anticonvulsants (*p* = 0.021)) were associated with lower scores chronically. Baseline depression (*p* < 0.001; *p* = 0.029) was associated with lower scores at both time points.	*p* < 0.02

## Data Availability

All data used in the results are included within the studies in the article.

## Abbreviations

OSA	Obstructive sleep apnea
SDB	Sleep-disordered breathing
AHI	Apnea–hypopnea index
SpO2	Oxygen saturation
Aβ	Amyloid beta peptide
CPAP	Continuous positive airway pressure
CVA	Cerebrovascular accident
PSD	Post-stroke depression
ODI	Oxygen desaturation index
HAMD	Hamilton Depression Scale
NO	Nitric oxide
